# Factors Associated With Recent HIV Infections Among Newly HIV Diagnosed in Rwanda

**DOI:** 10.1097/QAI.0000000000003669

**Published:** 2025-04-02

**Authors:** Eric Remera, Frédérique Chammartin, Sabin Nsanzimana, Samuel S. Malamba, Gallican N. Rwibasira, David J. Riedel, Jamie I. Forrest, Leon Mutesa, Nathan Ford, Ayman Ahmed, Jeanine U. Condo, Steven Karera, Edward J. Mills, Heiner C. Bucher

**Affiliations:** a Research Innovations and Data Science Division, Rwanda Biomedical Centre, Kigali, Rwanda;; b Division of Clinical Epidemiology, Department of Clinical Research, University Hospital Basel and University of Basel, Basel, Switzerland;; c Swiss Tropical and Public Health Institute, Basel, Switzerland;; d Ministry of Health, Kigali, Rwanda;; e Uganda Virus Research Institute (UVRI), Entebbe, Uganda;; f University of Maryland School of Medicine, Baltimore, MD;; g School of Population and Public Health, University of British Columbia, Canada;; h Centre for Human Genetics, College of Medicine & Health Sciences, University of Rwanda, Kigali, Rwanda;; i Centre for Infectious Disease Epidemiology and Research, School of Public Health and Family Medicine, University of Cape Town, Cape Town;; j Institute of Endemic Diseases, University of Khartoum, Khartoum, Sudan;; k School of Public Health, College of Medicine & Health Sciences, University of Rwanda, Kigali, Rwanda;; l Tulane University, New Orleans, LA; and; m Department of Health Research Methods, Evidence, and Impact, McMaster University, Hamilton, Canada.

**Keywords:** recency testing, partner notification services, HIV-1, Rwanda

## Abstract

**Introduction:**

Rwanda has initiated recency testing alongside existing HIV testing services of provider-initiated testing, partner notification services (PNSs), and of prevention of mother-to-child HIV transmission. We aimed to determine characteristics of the newly diagnosed population using a nationwide cohort.

**Methods:**

We included all newly diagnosed patients with HIV aged 15 and above who consented to recency testing and assessed patient- and health center–related predictors of recent HIV infection using multivariable logistic regression models.

**Results:**

We obtained data from 485 of 565 health facilities in Rwanda that introduced PNS and recency testing alongside preexisting testing services. From October 2018 to February 2024, 8940 individuals consented to HIV recency testing. Among them, 537 (6.0%) were recently infected. The odds of detecting a recent HIV infection increased by 1% for each month of experience in PNS. Patient-related factors associated with recent infections included female sex, younger age (15–24 years), residing in southern or northern provinces compared with the western province, and self-reported sex with a known HIV-infected person or unprotected sex outside of a relationship in the last 12 months.

**Conclusions:**

Only 6% of newly diagnosed HIV infections were characterized as recent. Public health interventions targeting younger females may assist in reducing new infections in this group.

## BACKGROUND

Early diagnosis of HIV and surveillance for recent HIV infections is crucial to understand and reduce HIV transmission through identifying at-risk groups with ongoing HIV transmission, categorizing risk factors for new infections, and timely linkage of newly infected individuals to HIV care and antiretroviral treatment.^1,2^

Recency-based incidence estimation using specific recency assays offers a practical and scalable method for HIV surveillance, particularly in high-burden or resource-limited settings.^3^ Differences between recency-based surveillance and observational study results stem from variations in their underlying methodologies, assumptions, such as the mean duration of recent infection and the false recency rate, and potential sources of bias. Although recency-based methods provide scalable and timely surveillance data, they necessitate careful calibration and interpretation to ensure consistency with findings from observational studies.

Integrating assays for recent HIV-1 infections into routine HIV testing services (HTS) for newly diagnosed cases could facilitate the development of an early warning and response system.^4^ This transformation of the current surveillance system would enhance HIV prevention and control efforts, improve care, and lead to better outcomes for patients through more targeted programmatic interventions.

The Joint United Nations Programme on HIV and AIDS (UNAIDS) 2022 estimates show that 45.8% of 1.2 million new HIV infections occur in the Global South.^5^ According to the Rwanda Population–based HIV Impact Assessment household survey in 2018–2019, the annual incidence of HIV is 0.08 per 100 person-years, yielding 5400 new infections annually for an adult population of 8.6 million.^2,6^ In addition, Rwanda Population–based HIV Impact Assessment estimated that 16% of all people living with HIV (PLHIV) (equivalent to 36,350 people) are not aware of their HIV status. From 2019 to 2023, around 11,000 individuals have been newly diagnosed and initiated on antiretroviral therapy (ART) every year.^7–10^

Rwanda added recency testing for individuals newly diagnosed with HIV in October 2018 to its HIV testing program for the detection of new HIV infections. This HIV testing program is voluntarily offered in 98% of all clinics across the country to all pregnant females and their partners, and to targeted individuals at risk of HIV acquisition. Starting in October 2018, an index testing program, referred to as partner notification services (PNSs), was gradually introduced in the health facilities, where index cases are encouraged to actively notify their partner themselves or through collaboration with health care providers.^9^ In this study, we aim to investigate the combined effect of public health interventions (partner notification and recency HIV testing) on disease surveillance and factors associated with recent HIV infections among individuals with a first positive HIV antibody test, which were detected in health facilities in Rwanda.

## METHODS

### Study Design and Setting

This is a cross-sectional study of individuals who were newly diagnosed with HIV in any of 485/565 (85.9%) health facilities across all the provinces of Rwanda, offering the full package of HIV testing, care, and treatment services for PLHIV. HTS include regular HTS, provider-initiated testing, prevention of mother-to-child HIV transmission (PMTCT) services (testing both pregnant women and their partners), or PNS. In PMTCT services, a pregnant woman is offered an HIV test at the first antenatal care visit and is retested at delivery if she was negative during antenatal care. Male partners are also tested whenever mothers are confirmed HIV positive at delivery. All individuals diagnosed with HIV are requested to consent to HIV recency testing and are offered immediate ART initiation, irrespective of their consent to recency testing.

The recency test and partner notification programs were initiated in October 2018 in 23 health facilities in Kigali, as a pilot project and were added in parallel to the preexisting HTS and PMTCT services. PNS are provided to individuals (index cases) with a newly diagnosed and known HIV infection^11^ to notify and detect undiagnosed HIV infections. Recency and partner notification programs were gradually scaled up across Rwanda from 2018 onward. By February 29, 2024, 565 health facilities offering HIV testing, care, and treatment services were trained in the provision of PNS and HIV recency testing and 485 centers provided data of newly diagnosed PLHIV.

### Study Population

The study population consists of individuals aged 15 years and above who were newly diagnosed with an HIV infection between October 1, 2018 and February 29, 2024, at all health facilities providing comprehensive HIV services, including index and recency testing. Only newly diagnosed participants who self-reported being unaware of their HIV-positive status and who had never been enrolled in ART treatment at any health facility were included in the study.

### Study Variables and Sources

The primary outcome is a recent HIV-1 infection diagnosis for individuals with a first documented HIV antibody positive test. Recent infection is diagnosed using the Asanté HIV-1 Rapid Recent Infection Assay, by Sedia Biosciences that detects infections within ≤12 months' duration, with an estimated false recency rate of about 1%.^12^ In addition to the Asanté assay, HIV viral load test is used according to the Recent Infection Testing Algorithm to further improve the accuracy of recent HIV diagnoses.^11,13,14^ The algorithm is defined such that a recent infection indicated by the Rapid Recent Infection assay has to be confirmed by a viral load >1000 copies/mL. Potential predictors of recent HIV infection were collected at health system and patient levels. Health system–related factors were HIV testing modality (HTS, provider-initiated testing, PMTCT, and PNS), time since health facilities initiated index testing defined as the difference in months between recency testing date and date when the health facility started index testing, number of patients enrolled on ART at health facility (<250, 250–500, 500–1000, 1000–1500 and 1500+), and the percentage of patients on ART with viral load below 200 copies/mL at health facility level (<95%, 95%–97%, 97%–98%, and above 98%). Individual patient factors were age (15–24, 25–34, 35–44 and 45+ years), sex, marital status (married or cohabitating/single, divorced, separated or widowed), residence (rural and urban), employment status (employed/unemployed), and self-reported HIV risk behavior in the last 12 months before HIV testing (sex with known HIV infected person/out of relationship unprotected vaginal sex/sex with multiple partners). The selection of potential predictors was based on literature review and HIV prevention expert opinion. At all health facilities, data from index testing and HIV recency are recorded into a case reporting form by a trained nurse, anonymized and keyed by a data manager into the electronic format of the District Health Information system, Version 2, a free, open-source health management data platform developed by the University of Oslo.^13^

### Statistical Analysis

We developed a multivariable logistic regression model to assess the association of individual and facility-related characteristics with the risk of HIV recent infection in newly diagnosed individuals. Because recency testing is not consistently offered across province and health facilities, we weighted our analysis with weights defined as the product of the inverse of the proportion of health facilities providing recency testing services at the province level and the inverse of the proportion of newly diagnosed cases who were offered HIV recency tests within each health facility. Hence, we created a pseudo-population where individuals seen in provincial and health facility where recency testing is less commonly offered are upweighted. The chosen approach aims at balancing covariates and reduce selection bias. Adjusted odds ratios (aORs) with 95% confidence intervals (CIs) are presented, and statistical significance was determined by whether the 95% CIs exclude 1.00 or *P*-value >0.05.

All analyses were done in R statistics, version 4.1.3.

## RESULTS

**FIGURE 1.**
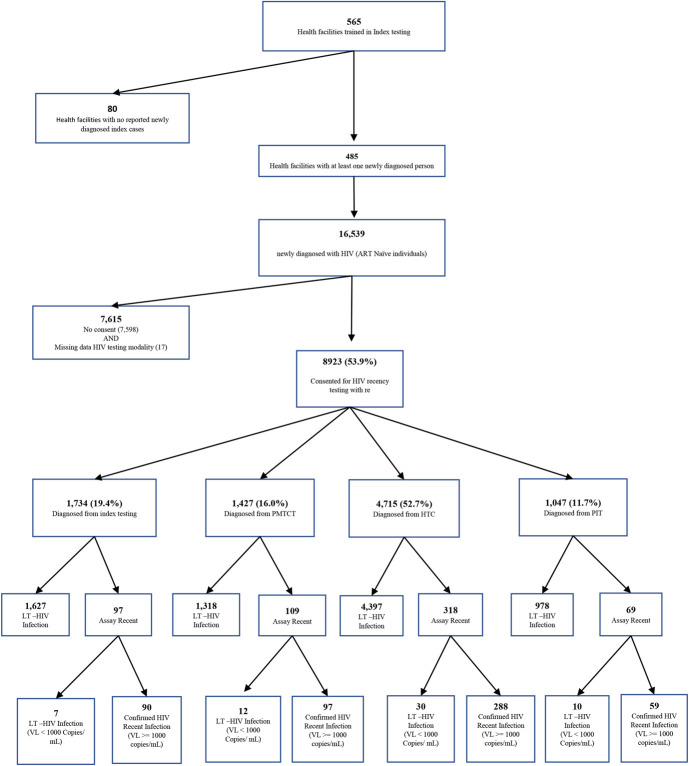
Recruitment flow.

Between October 1, 2018, and February 29, 2024, a total of 16,539, naïve patients diagnosed with HIV from 485 health facilities, initiated ART. Of them, 8940 (54.1%) consented to HIV recency testing and were included in our analysis (Fig. 1).

Participant characteristics, together with associated percentages of recent infection, are presented in Table 1. Median age was 32.9 (interquartile range 26.5–40.2), 5.778 (64.6%) were female, 6.119 (68.4%) resided in rural areas, 4496 (50.3%) were married or cohabitating, and 1561 (17.5%) engaged in a sexual relationship with known HIV-positive partners. PNS led to the diagnosis of 1734 individuals (19.3%). Health facilities had a median experience in index and recency testing of 25 months (interquartile range: 14–36).

**TABLE 1.** Study Participant's Characteristics and Percentage of HIV Recent Infections Among Individuals With a First Positive HIV Antibody Test Between October 1, 2018 and February 29, 2024 in Rwanda

	Study Participants, n = 8940		HIV Recent Infections	
	n	%	n	%
Median age (IQR) (yrs)	32.9 (26.5–40.2)			
Age (yrs)				
15–24	1747	19.7	159	9.1
25–34	3433	38.1	189	5.5
35–44	2428	27.1	126	5.2
45+	1332	15.1	63	4.7
Sex*				
Female	5778	64.6	397	6.9
Male	3161	35.3	140	4.4
Province*				
East	2769	29.7	161	6.1
City of Kigali	3230	37.9	185	5.5
North	561	6.0	53	9.9
South	1091	12.2	84	7.7
West	1289	14.3	54	4.2
Residence				
Rural	6119	68.3	377	6.2
Urban	2821	31.7	160	5.6
Occupation*				
Not employed	4669	52.7	232	6.5
Employed	4259	47.3	305	5.5
Living status				
Single	2941	33.1	185	6.3
Married/cohabitant	4496	50.3	272	6.1
Separated/divorced/widow	1494	16.7	80	5.4
HIV testing modalities*				
Index testing	1734	19.3	90	5.2
PMTCT	1427	15.6	97	7.0
HTC	4715	53.4	288	6.0
PIT	1047	11.6	59	5.7
Median time since start of index testing at health facility (IQR) (mo)	25 (14–36)			
Facility size according to number of patients on antiretroviral				
<250	1179	13.6	108	8.9
250–500	1846	20.0	103	5.8
500–1000	2838	31.0	165	6.0
100–1500	1307	14.9	72	5.4
>1500	1770	20.5	88	4.8
Viral suppression percentage of health facility				
below 95%	699	7.8	53	7.7
95%–97%	1727	19.6	130	7.4
97%–98%	2528	29.2	147	5.6
Above 98%	3986	43.5	207	5.3
Sexual behavior risks				
Unprotected sex in the last 12 mo*				
No	971	11.1	42	4.2
Yes	7935	88.9	495	6.2
Sex with multiple partners*				
No	5365	59.9	316	5.9
Yes	3561	40.1	221	6.2
Sex with known HIV-positive partner(s)*				
No	7363	81.9	405	5.5
Yes	1561	18.1	132	8.1

* Variables with missing data.

HTC, HIV testing and counseling; IQR, interquartile range; PIT, provider initiated testing.

Recent HIV infection was confirmed in 537 (6.0%) individuals in this study. A higher proportion of recent infections were observed among individuals aged between 15 and 24 years [159 (9.1%)], females [397 (6.9%)], residents of the northern province [53 (9.9%)], individuals diagnosed through PMTCT services [97 (7.0%)], and individuals who self-reported engaging in sexual intercourse with an HIV-positive partner [132 (8.1%)]. Furthermore, HIV recency rate was higher in PLWH visiting health facilities with less than 250 PLWH on ART [108 (8.9%)], as well in PLWH seen in health facilities with a low viral suppression rate below 95% [53 (7.7%)] (Table 1).

Results of multivariable analyses of the risk of being recently infected with HIV in newly diagnosed PLWH are shown in Table 2. Young individuals aged 15–24 years old compared with those aged 45 years and above [aOR 1.91; 95% CI: 1.33 to 2.75], female (aOR 1.47; 95% CI: 1.13 to 1.92) were at increased risk of recent HIV infection. Participants who reported having sex with an HIV-positive partner (aOR 1.59; 95% CI: 1.24 to 2.03) and those who reported having had out-of-relationship unprotected vaginal sex in the last 12 months (aOR 1.58; 95% CI: 1.09 to 2.31) showed an increased risk of HIV recency. In comparison to the western province of Rwanda, individuals living in the northern and southern provinces were also positively associated to the risk of recent infection (aOR 2.01; 95% CI: 1.08 to 3.73 and aOR 1.82; 95% CI: 1.07 to 3.10, respectively). Further, at each increase in time since the health facility started to implement index testing, the likelihood of detecting recent infections increase by 1% (aOR 1.01; 95% CI: 1.00 to 1.02) (Table 2).

**TABLE 2.** Univariable and Multivariable Analyses of the Risk of Being Recently Infected With HIV in Newly Diagnosed PLHIV

	Bivariate Analysis		Multivariable Analysis	
	OR	95% CI	aOR	95% CI
Age group				
15–24	2.06	1.5 to 2.81	1.91	1.33 to 2.75
25–34	1.19	0.87 to 1.61	1.15	0.81 to 1.64
35–44	1.12	0.81 to 1.55	1.14	0.8 to 1.63
45+	1.00		1.00	
Gender				
Male	1.00		1.00	
Female	1.46	1.2 to 1.79	1.47	1.13 to 1.92
Province				
West	1.00		1.00	
East	1.38	1 to 1.91	1.40	0.84 to 2.33
Kigali City	1.30	0.94 to 1.79	1.15	0.61 to 2.17
North	2.43	1.62 to 3.63	2.01	1.08 to 3.73
South	1.87	1.3 to 2.68	1.82	1.07 to 3.10
Residence				
Urban	1.00			
Rural	1.08	0.91 to 1.29		
Occupation				
Not employed	1.00		1.00	
Employed	1.17	0.97 to 1.41	1.18	0.94 to 1.47
Living status				
Single/separated/divorced/widow	1.00			
Married/cohabitant	1.02	0.84 to 1.21		
HIV testing modalities				
Index testing	1.00		1.00	
PMTCT	1.42	1.05 to 1.93	1.13	0.80 to 1.60
HTC	1.20	0.93 to 1.55	1.16	0.85 to 1.59
PIT	1.13	0.8 to 1.61	1.05	0.66 to 1.66
Time since start if index testing at health facility [median (SD)]	1.00	1 to 1.01	1.01	1.00 to 1.02
Number of patients on antiretroviral at health facility				
<250	2.12	1.56 to 2.9	1.46	0.80 to 2.69
250–500	1.24	0.91 to 1.7	0.96	0.51 to 1.81
500–1000	1.37	1.03 to 1.82	1.07	0.55 to 2.08
100–1500	1.18	0.84 to 1.66	1.00	0.50 to 2.02
>1500	1.00		1.00	
Viral load suppression (<1000 copies/mL) rate at health facility				
Below 95%	1.49	1.08 to 2.06	1.40	0.79 to 2.48
95–97	1.37	1.08 to 1.73	1.57	0.99 to 2.46
97–98	1.07	0.86 to 1.35	1.18	0.75 to 1.86
98+	1.00		1.00	
Sexual bahavior risks				
Had any unprotected sex in last 12 mo (ref:no)	1.37	0.99 to 1.9	1.58	1.09 to 2.31
Had sex with multiple partners (ref:no)	1.10	0.92 to 1.32	1.01	0.79 to 1.29
Had sex with known HIV positive partner(s) (ref:no)	1.57	1.27 to 1.94	1.59	1.24 to 2.03

HTC, HIV testing and counseling; PIT, provider initiated testing.

## DISCUSSION

Our findings reveal risk factors and demographic characteristics of population groups at high risk of new HIV infection. The overall percentage of HIV recent infections among study participants was 6.0%, indicating that most newly diagnosed PLWH have been infected more than 1 year ago. Although recent cases (6%) are more infectious than long-term cases (94%), the latter may continue to transmit the virus over a longer period.

Our results also showed that health facilities with greater than 6 months' experience in index testing had lower rates of recent infections (3.9%, 138/3528) compared with facilities with less than 6 months' experience in index testing (5.1%, 70/1383; aOR 0.73, 95% CI: 0.61 to 0.86), possibly because of improvements in staff expertise and enhanced patient engagement.

Studies conducted in Kenya and Zimbabwe also reported a low percentage of recent infections among newly diagnosed individuals.^9,15,16^ We found that adolescents and young adults (aged 15–24 years) were at a higher risk of recent infection compared with adults older than 45 years. Similar trends have been observed in various studies across sub-Saharan Africa, reporting that over 35% of new infections are occurring among adolescents and young adults.^17,18^ This might be attributed to the young population tendency for having risky behaviors including unprotected sex and multiple partners.^19^

Our findings also showed that females were more likely to consent to a recency test and were more likely to have a recent infection than males (Table 1). Similar results are reported in studies conducted previously in Rwanda and in South Africa.^20–22^ This finding should urge for improving the community engagement and HIV counseling to engage the male population at risk. The last Rwanda demographic health survey estimated that 78% of adult females have been tested compared with 64% of males.^21^ In addition, women of childbearing age are likely to have regular gynecological follow-ups and as a consequence to be more frequently offered an HIV test.^12,23–25^ Testing of partners of pregnant women before an HIV diagnosis is crucial, particularly given the risk of HIV acquisition during pregnancy and the potential for mother-to-child transmission in cases of acute infection.

The results also found that individuals who self-reported engaging in sexual activity with known HIV-positive partners and those reporting unprotected sex were more likely to have recently acquired HIV. This highlights the role of recency testing in the early detection and prevention of further spread of HIV among the female and youth populations. Further research is warranted to better understand the characteristics of individuals engaging in unprotected sex and sexual relationships with known HIV-positive partners and the importance of reinforcing access to pre- and postexposure prophylaxis, especially for pregnant women and women of childbearing age.

Strengths of our study include its contemporary insight into recency of infection^26^ in Rwanda with national coverage and data of around 9000 newly diagnosed PLWH over the last 6 years. The novelty and feasibility to upscale this service offers a great opportunity to improve disease surveillance and response through the early detection and enrolment in treatment of recent infections. Furthermore, this strategy has helped in identifying risk factors associated with recent HIV infection. Considering the rapid advancement in diagnostic tools development, this strategy could be expanded to monitor the dynamic and transmission of other infectious diseases.^27^ However, 1 major challenge for the full deployment and uptake of recency testing is that it is a voluntary test for individuals newly diagnosed with HIV and the refusal rate for recency testing was relatively high. This could be addressed through enhancing community engagement to change behavior. A limitation of our study includes the precision of the recency testing. There is an ongoing debate about the accuracy and reliability of the recency testing assays. One study from Uganda among ART naïve patients with known HIV seroconversion dates reported an excellent specificity ranging from 87% to 100%, whereas sensitivity was relatively low at 31.8% and 49.4%.^26^ Another study among newly diagnosed pregnant adolescent girls and young women in Malawi and a household-based survey in Swaziland also found excellent sensitivity in addition to a high correlation with the limiting antigen avidity enzyme immunoassay used in recent infection testing algorithms.^,14^ Our study combined the assay with the viral load as it improves the accuracy of the recency test.^14,28^

In conclusion, our study showed a small increase in detecting recent infections at health facilities gained experience with index testing. The study demonstrated the value of identifying recent infections and associated risk factors. Scaling up recency testing services and continuous surveillance could be an important step forward for monitoring ongoing transmissions and an opportunity for prevention planning.
